# Proteins enriched in charged amino acids control the formation and stabilization of selenium nanoparticles in *Comamonas testosteroni* S44

**DOI:** 10.1038/s41598-018-23295-5

**Published:** 2018-03-19

**Authors:** Ding Xu, Lichen Yang, Yu Wang, Gejiao Wang, Christopher Rensing, Shixue Zheng

**Affiliations:** 1 State Key Laboratory of Agricultural Microbiology, College of Life Science and Technology, Huazhong Agricultural University, Wuhan, 430070 P. R. China; 2 Institute of Environmental Microbiology, College of Resources and Environment, Fujian Agriculture & Forestry University, Fuzhou, Fujian 350002 P. R. China

## Abstract

Elemental selenium nanoparticles (SeNPs) are useful in medicine, environmental remediation and in material science. Biosynthesized SeNPs (BioSeNPs) by bacteria are cheap, eco-friendly and have a lower cytotoxicity in comparison with chemically synthesized ones. Organic matters were found to cap on the surface of BioSeNPs, but the functions were still not entirely clear. The purified BioSeNPs were coated in a thick layer of organic substrates observed by transmission electron microscopy (TEM). Fourier Transform Infrared (FT-IR) and quantitative detection of the coating agents showed that one gram of purified BioSeNPs bound 1069 mg proteins, 23 mg carbohydrates and only very limited amounts of lipids. Proteomics of BioSeNPs showed more than 800 proteins bound to BioSeNPs. Proteins enriched in charged amino acids are the major factor thought to govern the formation process and stabilization of BioSeNPs in bacteria. In view of the results reported here, a schematic model for the molecular mechanism of BioSeNPs formation in bacteria is proposed. These findings are helpful for the artificial green synthesis of stable SeNPs under specific condition and guiding the surface modification of SeNPs for medicine application.

## Introduction

Selenium (Se) is an essential trace element in humans and many microorganisms with a broad utility in biological systems^1^. Selenocysteine (the 21th amino acid) constitutes the active center of 25 selenoproteins^2^. Se deficiency can lead to many diseases such as Kashin-Beck^3^, cognitive impairment, seizures, Parkinson’s disease and Alzheimer’s disease^4^, and also to gastrointestinal and thyroid problems^5^. Se exists in four states (−2, 0, +4 and +6) with chemical forms of selenide, elemental selenium, selenite and selenate. Elemental selenium nanoparticles (SeNPs) exhibited low cytotoxicity compared to other selenium compounds with different valence state^6,7^. In addition, SeNPs displayed excellent anticancer and therapeutic activities, anti-biofilm, anti-oxidant, wound healing, cytotoxic and anti-viral activities in medical application^8–12^. SeNPs as a carrier of medicine exhibit a great potential in the future application^13,14^. SeNPs also have been used in other fields, such as heavy metal removal processes^15^ and improvement of medical materials^16^. Accordingly, SeNPs present a great potential for applications in medicine, remediation and material sciences.

The process of biosynthesizing selenium nanoparticles (BioSeNPs) is safe and cheap and employs eco-friendly non-toxic materials^17^. In contrast, physicochemical methods to synthesize SeNPs may render the nanoparticles unsafe for biomedical applications due to the unfavorable reaction conditions, such as high temperature, acidic pH, and harsh chemicals^18^. Diverse bacteria and fungi synthesize SeNPs through reduction of Se oxyanions (selenite and selenate)^19–21^. BioSeNPs exhibited low cytotoxicity in comparison with chemically synthesized SeNPs^22^. Synthesis of SeNPs by bacteria was shown to take place in the cytoplasm, the periplasm or/and extracellular spaces^20,23–25^, suggesting the mechanisms of SeNPs formation are variable. In the environment, the size and colloidal property of BioSeNPs (20–500 nm) governed their transport and fate^26^. The bioremediation efficiency and nanotoxicological aspects such as dissolution and surface reactivity are also influenced by these properties^27–29^.

Proteins are found to associate with BioSeNPs^30–32^, but the functions are still unclear. In particularly, a specific protein SefA is found to associated on BioSeNPs may play the role of assembling the BioSeNPs in an anaerobic bacterial strain^33^, but it is difficult to find a protein of similar function in other aerobic bacteria^28,31^. Furthermore, extracellular polymeric substances (EPS) governed the surface charge of BioSeNPs and made them stable in colloidal suspensions^26^. However, the colloidal property, formation and stabilization of BioSeNPs intracellular are still not understood. Thus, it is of great significance to better understand the processes leading to the formation and stabilization of BioSeNPs in bacteria. This would be helpful for mass green production on an industrial scale and guiding surface modification of SeNPs for medicine application.

In this study, BioSeNPs were produced by *Comamonas testosteroni* S44 which reduced Se (IV) to red-colored elemental selenium nanoparticles (BioSeNPs) under aerobic condition. Then BioSeNPs were extracted and their colloidal properties were analyzed quantitively to understand the factors governing the formation and stabilization of BioSeNPs. It suggests that intracellular organic matter especially on proteins are the capping agents and thus affect the surface charge of the BioSeNPs and its stability and non-specified functional but charged amino acid enriched proteins control the formation and stabilization of the selenium nanoparticles.

## Results

### Formation of BioSeNPs in *C. testosteroni* S44

**Figure 1:**
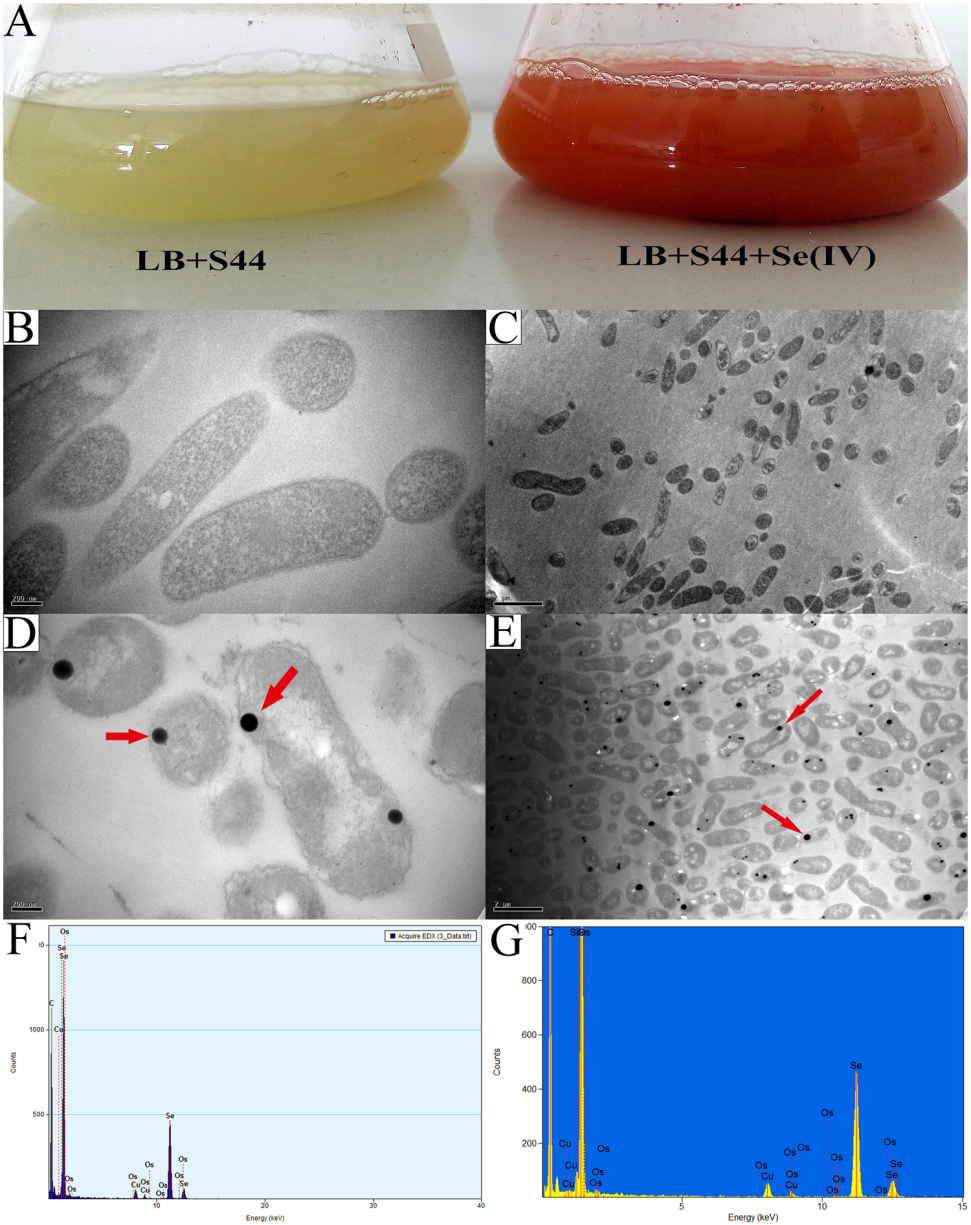
*Comamonas testosteroni* S44 reduced selenite to red elemental SeNPs. Growth of S44 on LB broth with 10.0 mM sodium selenite and control (**A**). TEM images of SeNPs (**D** and **E**) and controls (**B** and **C**), and EDX spectra of BioSeNPs.

*C. testosteroni* S44 reduced Se (IV) to red-colored elemental selenium nanoparticles (SeNPs) under aerobic condition in LB broth (Fig. 1A). The SeNPs were not observed in cells growing in lower concentrations of Se (IV) (1.0 mM)^34^. In contrast, SeNPs occurred in most cells when the Se (IV) concentration was elevated to 10 mM as shown by TEM (Fig. 1D and E) and X-ray spectroscopy (EDX) (Fig. 1F and G). The EDX spectrum revealed the presence of three selenium peaks of SeLα, SeKα, and SeKβ transitions at 1.37, 11.22 and 12.49 keV, respectively. It was interesting that most of the intracellular SeNPs were in proximity of the cell border whereas only a few SeNPs were located inside the cytoplasm (Fig. 1E). The cell and outer membrane of *C. testosteroni* S44 with added Se (IV) became discrete and corrugated compared to the control (Fig. 1B and D). These results indicated that the most of the Se (IV) reduction process and subsequent BioSeNPs formation occurred closing to the inner membrane with only of few occurring in the cytoplasm. At a later stage, the SeNPs may transfer to the extracellular space by cell lysis.

### Characterization of BioSeNPs and CheBioSeNPs

**Figure 2:**
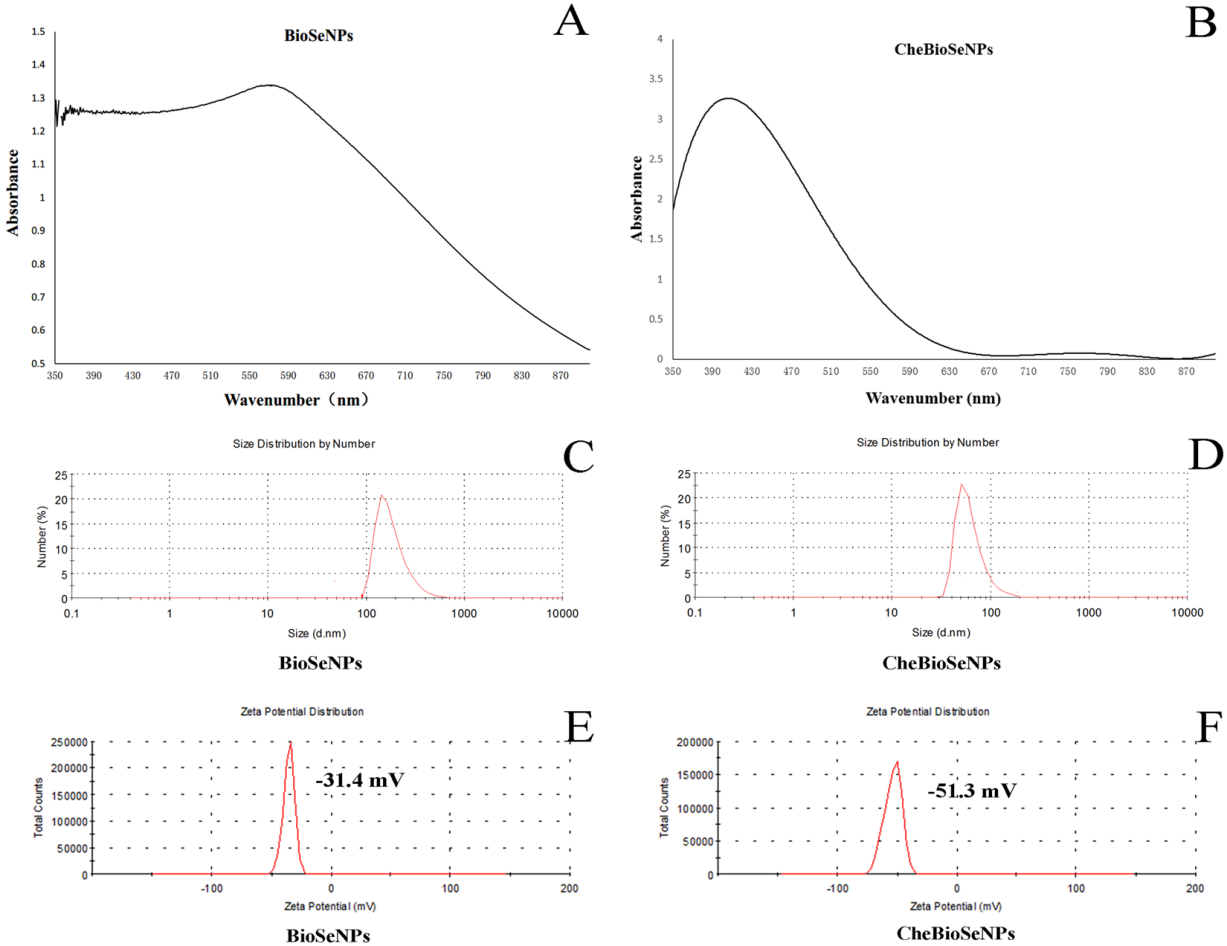
Absorption peak (**A** and **B**), size distribution (**C** and **D**) and zeta potential (**E** and **F**) of SeNPs. BioSeNPs, SeNPs produced in *C. testosteroni* S44 and then be purified. CheBioSeNPs, glutathione-reduction-synthesized SeNPs under cellular fractions.

In order to understand the factors affecting aggregation of elemental selenium to BioSeNPs, the SeNPs generated by *C. testosteroni* S44 cells were purified by sonication and centrifuged with 80% (w/v) sucrose (Fig. S1). Meanwhile, glutathione-reduction-synthesized SeNPs (CheBioSeNPs) under the reaction system of cellular fraction were also generated *in vitro* as control. The purified BioSeNPs and CheBioSeNPs were collected and suspended in ddH_2_O for visible light spectrum scanning (350–900 nm). BioSeNPs and CheBioSeNPs showed a maximum absorption peak at 572 nm and 412 nm, respectively (Fig. 2A and B). This difference corresponded to their size distribution^35^, and the maximum absorption of BioSeNPs varied in different bacteria^36,37^. Dynamic Light Scattering (DLS) analysis showed that the average size of BioSeNPs and CheBioSeNPs was 252 nm and 96 nm, respectively (Fig. 2C and D). The size range of most BioSeNPs and CheBioSeNPs was 100–300 nm and 30–100 nm, respectively. However, a few SeNPs displayed an extremely big size resulting in an increase of the average size. The unusually big size was probably due to a further aggregation of small particles. Zeta potential analysis showed BioSeNPs and CheBioSeNPs have a negative potential of 31.4 ± 3 mV and 51.3 ± 2 mV, respectively in ddH_2_O (Fig. 2E and F), which was similar to previous reports^26,32^.

**Figure 3:**
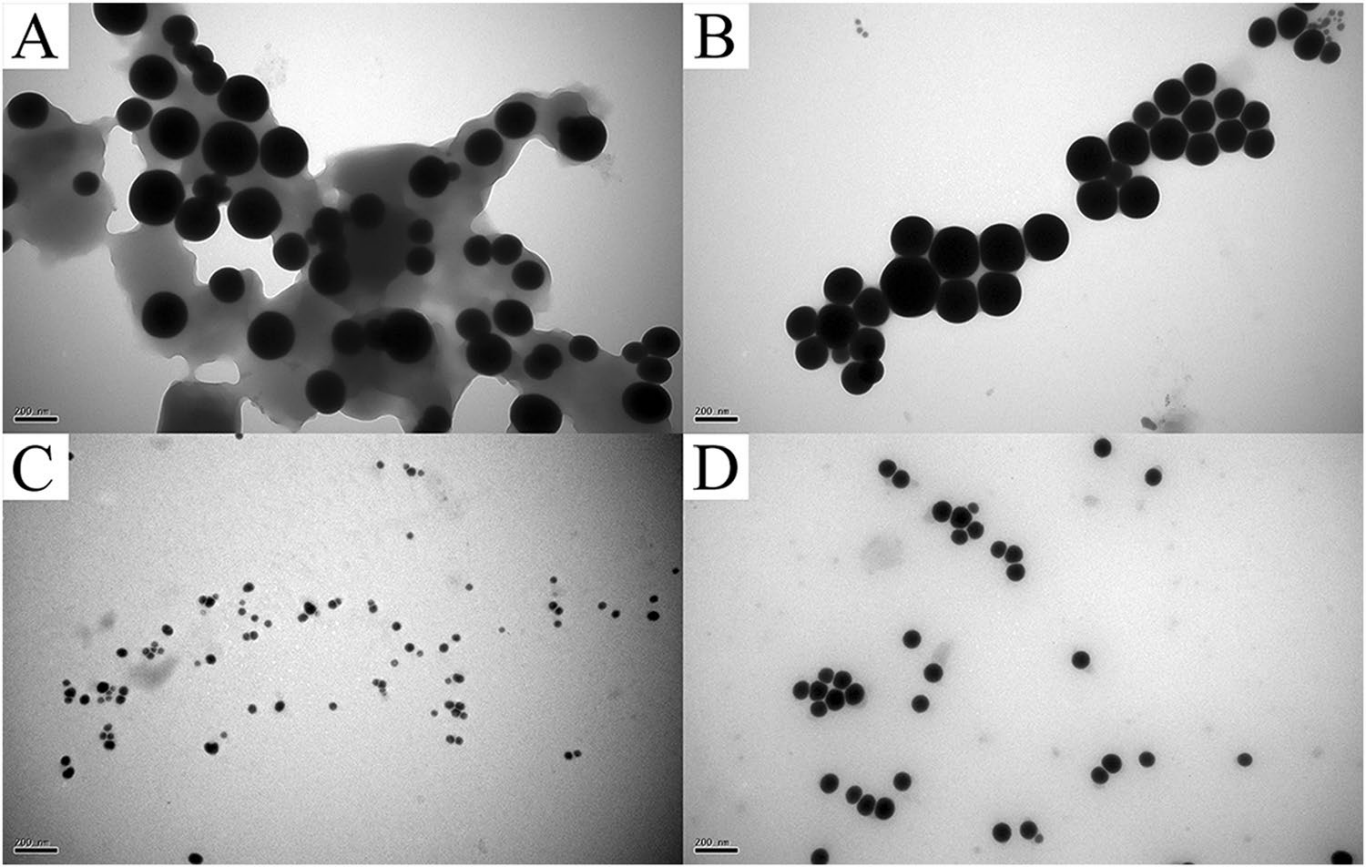
TEM images of organic agents coated and removed SeNPs. Agents coated (**A**) and removed BioSeNPs (**B**), agents coated (**C**) and removed CheBioSeNPs (**D**), respectively.

When the purified BioSeNPs synthesized by *C. testosteroni* S44 were visualized by TEM, we observed that the surface of spherical BioSeNPs were coated with a thick layer of organic matter (Fig. 3A). After being suspended in 10% SDS solution and incubated in boiled water for 20 min, most of the organic layer capping BioSeNPs was removed (Fig. 3B), but a few organic substrates were still adhering to the BioSeNPs despite SDS treatment and boiling (Fig. 3B). There was no difference in size between BioSeNPs after removing the coat and coated ones (Fig. 3A and B), but the SeNPs without an organic layer were a little easier to precipitate on the bottom of the container. Simultaneously, the CheBioSeNPs synthesized *in vitro* were also treated under the same condition. In contrast, the thin coating agents on CheBioSeNPs were almost completely removed and thus the size of CheBioSeNPs after removing coated agents (about 90–120 nm in diameter) grew bigger than coated ones (Fig. 3C and D).

### The agents coating on the BioSeNPs and CheBioSeNPs analyzed by FT-IR

**Figure 4:**
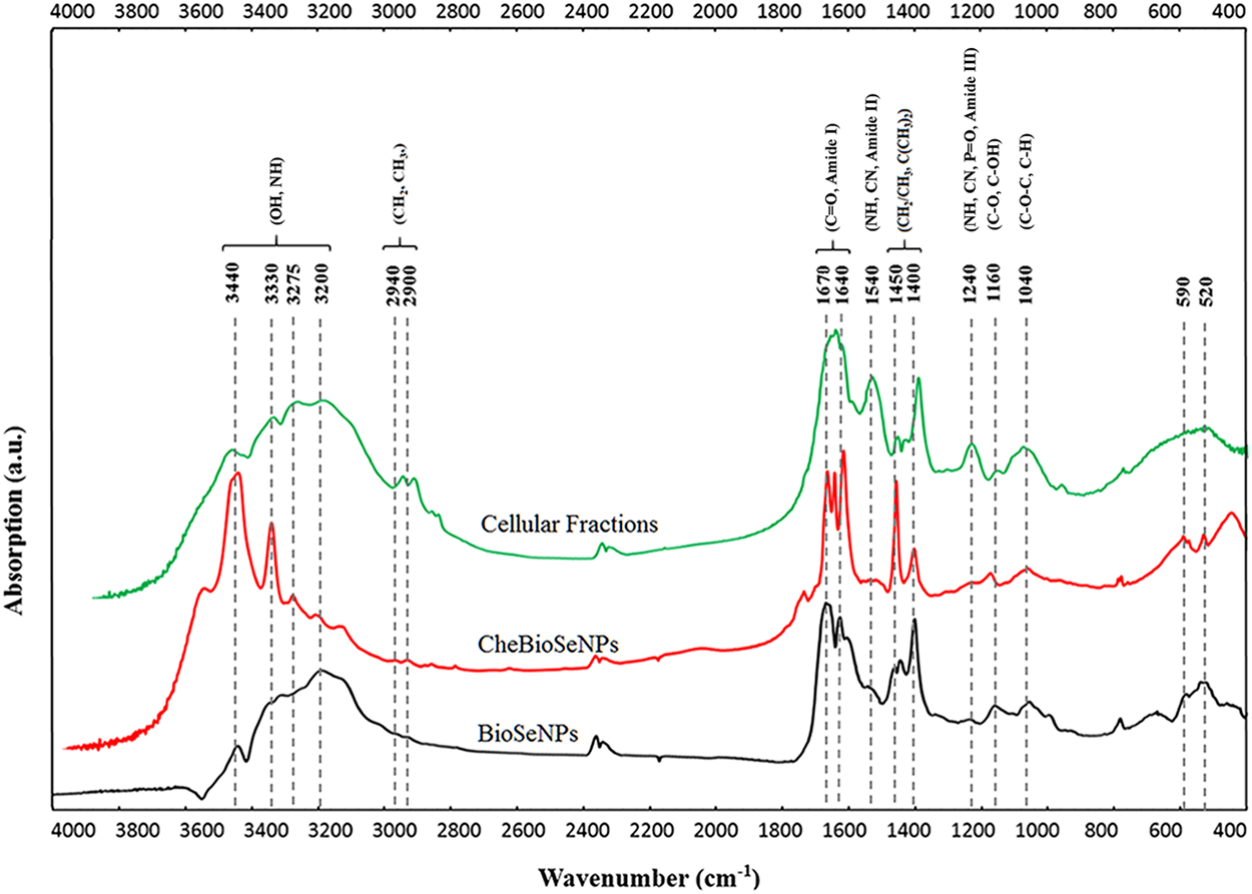
FT-IR spectra of cellular fractions, CheBioSeNPs and BioSeNPs and corresponding substrates. Indicated values are in cm^−1^.

To understand what organic substrates affected the formation of intracellular SeNPs, fourier transform infrared (FT-IR) spectroscopic analysis was performed (Fig. 4). Both BioSeNPs and cellular fractions displayed a broad feature between 3440 and 3200 cm^−1^, representing –OH, −NH stretching of protein, carbohydrates and lipids. The features at 2900 and 2940 cm^−1^ correspond to CH_2_ and CH_3_ stretching from lipids and proteins^26,38^. The strongest and sharp features especially on BioSeNPs at 1670 and 1640 cm^−1^, represents the stretching vibration of C=O present in proteins (amide I)^26,38^. The feature at 1540 cm^−1^ corresponded to the N–H and C–N vibrations of the peptide bond in different protein conformations (amide II)^26,39^. Another region between 1450 and 1400 cm^−1^ includes sharp features of BioSeNPs and CheBioSeNPs that were assigned to CH_2_/CH_3_ and C(CH_3_)_2_ stretching mainly in proteins and lipids^39^. The C−N stretching and N−H bending vibrations were also observed at 1240 cm^−1^ (amide III)^26^. On the other hand, the peak at 1240 cm^−1^ may also correspond to the *ν*_asym_ PO_2_^−^ in DNA, RNA and phospholipids^39^. The small features of BioSeNPs at 1040 cm^−1^ corresponding to C−O−C and C−H, and at 1160 cm^−1^ corresponding to C–O, C–OH, represented carbohydrates^26,39^. In summary, the overall shape of the FT-IR spectra confirmed the presence of proteins, carbohydrates and lipids on the surface of BioSeNPs.

### Comparison of coating proteins on the surface of BioSeNPs and CheBioSeNPs analyzed by SDS-PAGE

**Figure 5:**
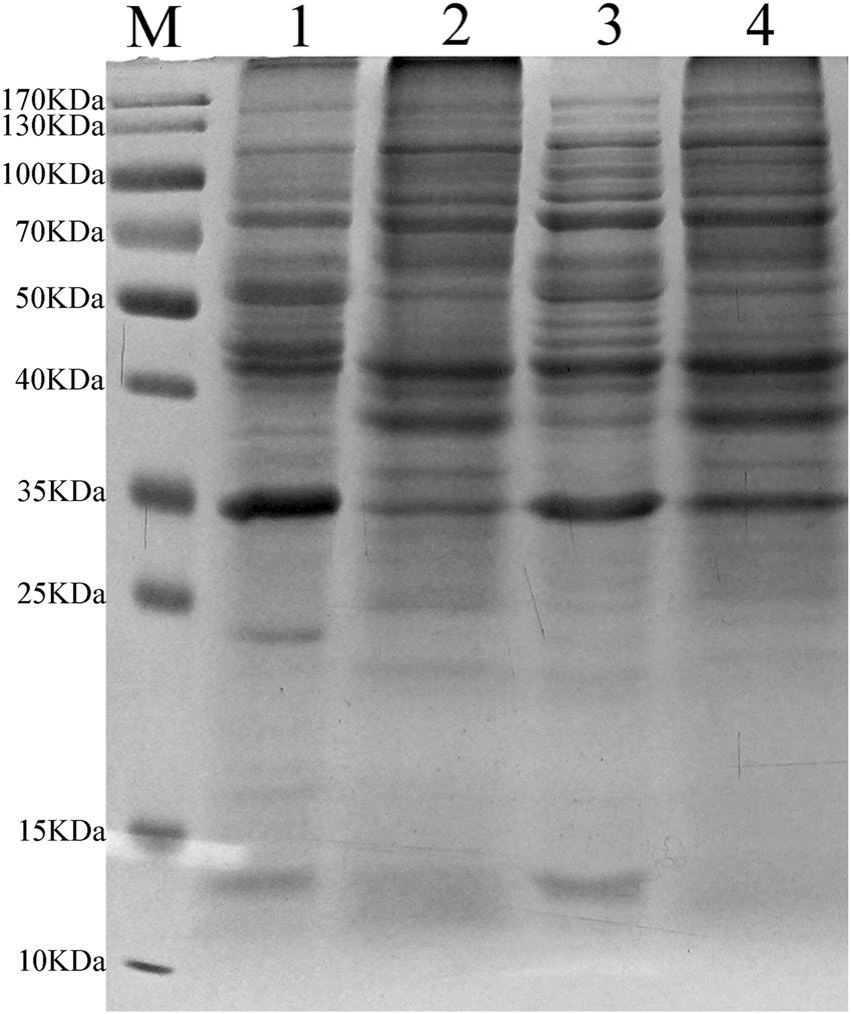
SeNPs coated proteins and corresponding proteins of SeNPs-producing environment by SDS-PAGE. M, marker, Lane 1, BioSeNPs coated proteins, lane 2, cellular proteins, lane 3, CheBioSeNPs coated proteins, lane 4, proteins of supernatant of producing CheBioseNPs.

After detection of components of coating agents by FT-IR analysis, the amounts of proteins, carbohydrates and lipids coating BioSeNPs were quantitatively determined respectively. The BioSeNPs produced from *C. testosteroni* S44 bound 1069 mg proteins and 23 mg carbohydrates on one gram of BioSeNPs. However, the lipids could not be quantified due to the limited amounts. The result indicated that proteins play a primary role in controlling the formation of BioSeNPs. Therefore, a 10% SDS solution was used to separate the proteins bound to BioSeNPs and CheBioSeNPs, and subsequently the proteins were analyzed by SDS-PAGE gel (Fig. 5). The protein profile of BioSeNPs (lane 1) was very similar with protein profile of total cellular proteins (lane 2) except for few differing bands. Likewise, similar protein profiles were observed in CheBioSeNPs (lane 3) and cellular fractions used to produce SeNPs *in vitro* (lane 4). Furthermore, protein bands almost showed no difference between BioSeNPs *in vivo* and CheBioSeNPs *in vitro*. These results indicated that diverse cellular proteins bound to the surface of SeNPs both *in vivo* and *in vitro*.

**Figure 6:**
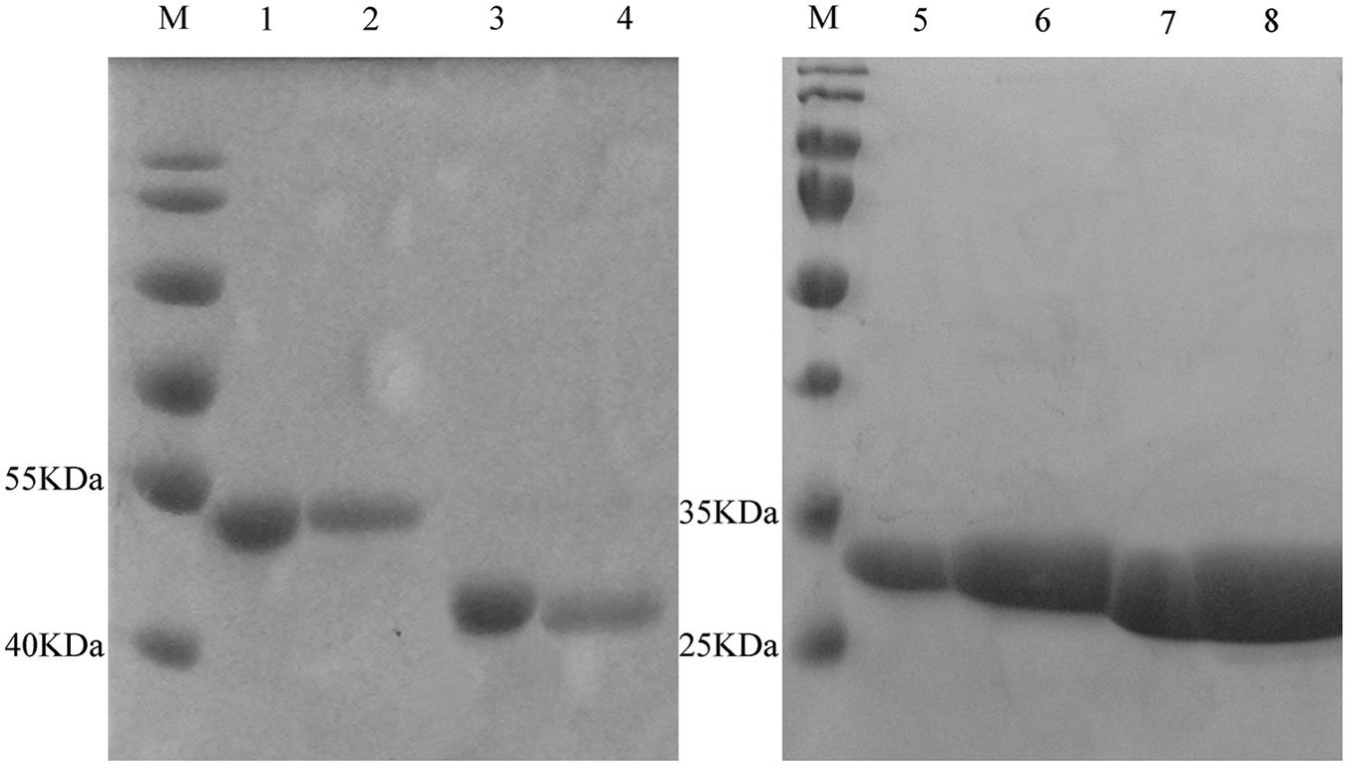
Comparison of four single proteins and coating state on SeNPs. M, marker, Lane 1 and 3, purified proteins Mop and CysB from strain S44, lane 2 and 4, SeNPs coated with Mop and CysB, lane 5 and 7, purified proteins PhoB1 and PhoB2 from *Agrobacterium* strain GW4, lane 6 and 8, SeNPs coated with PhoB1 and PhoB2.

It appears binding proteins were nonspecific and only resulted from physical-chemical reactions. To demonstrate this point, a protein binding assay was conducted for testing whether any protein had the ability to bind BioSeNPs. Accordingly, randomly selected and purified proteins Mop and CysB cloned from *C. testosteroni* S44, and PhoB1 and PhoB2 cloned from an *Agrobacterium* strain GW4 were added into the solution used for production of CheBioSeNPs respectively. Then CheBioSeNPs were examined to determine which proteins could be detected by SDS-PAGE as shown in Fig. 6. It showed that purified single protein strongly bound to SeNPs.

### Proteomics assay of BioSeNPs

A further proteomics assay investigation was quantitatively performed looking at the coating proteins on BioSeNPs. A multitude of 888 proteins with diverse functions were identified (Table S1). Meanwhile, cellular proteins of *C. testosteroni* S44 were analyzed as control with 826 proteins being identified (Table S2). Proteins present in more than 1% abundance were shown in Table 1. The most abundant peptides coating BioSeNPs were chaperone protein DnaK (6.1%), followed by elongation factor Tu (4.3%), as well as 10 other proteins encompassing zinc-dependent alcohol dehydrogenase, chaperone protein GroEL, porin, elongation factor G, citrate synthase I, universal stress protein A (UspA), ribosomal protein S1, electron transfer flavoprotein beta-subunit, heat shock protein 90 and succinyl-CoA synthetase beta-subunit. In contrast, the most abundant peptides present on the cellular proteins were porin (3.5%), followed by UspA (3.5%), and other 6 proteins. It was obvious that bound proteins on BioSeNPs did not completely match the cellular proteins and contents despite many common proteins were shared between them.

**Table 1:** Comparison of dominant BioSeNPs coating proteins and cellular proteins based on proteomics assay.

	Mass	Matches	emPAI^a^	Percent in coated proteins	Percent in cellular proteins	Charge (%)^b^	Protein annotation
BioSeNPs coating proteins	68903	822 (552)	38.52	6.10	0.44	**29.12**	chaperone protein DnaK [*C. testosteroni* CNB-2]
	41840	730 (535)	27.36	4.34	1.28	**26.01**	elongation factor Tu, partial [*Comamonas*]
	36645	341 (232)	19.71	3.12	0.27	**18.9**	zinc-dependent alcohol dehydrogenase [*C.testosteroni*]
	57066	466 (302)	16.46	2.61	1.84	**25.59**	chaperonin GroEL [*C. testosteroni* CNB-2]
	36960	191 (84)	13.3	2.11	3.52	**18.29**	porin [*C. testosteroni* S44]
	79505	283 (164)	9.02	1.43	0.43	**27.35**	elongation factor G [*C. testosteroni*]
	48996	120 (70)	8.79	1.39	0.39	**21.1**	citrate synthase I [*C. testosteroni* CNB-2]
	16234	35 (21)	8.76	1.39	3.51	**23.13**	universal stress protein (UspA) [*C. testosteroni* CNB-2]
	61312	268 (175)	7.09	1.12	0.23	**28.39**	ribosomal protein S1 [*C. testosteroni* KF-1]
	25585	67 (41)	7.08	1.12	0.34	**27.61**	electron transfer flavoprotein beta-subunit [*C. testosteroni* S44]
	72292	264 (147)	6.72	1.06	0.04	**29.28**	heat shock protein 90 [*C. testosteroni* S44]
	41414	126 (58)	6.37	1.01	0.26	**24.35**	succinyl-CoA synthetase, beta subunit [*C. testosteroni* CNB-2]
cellular proteins	**Mass**	**Matches**	**emPAI** ^**a**^	**Percent in cellular proteins**	**percent in coating proteins**	**Charge (%)** ^**b**^	**Protein annotation**
	36960	127 (76)	13.3	3.53	2.11	**18.29**	porin [C. testosteroni S44]
	16234	34 (22)	13.27	3.52	1.39	**23.13**	UspA [C. testosteroni CNB-2]
	21023	49 (28)	6.99	1.86	0.09	**20.94**	peroxiredoxin [C. testosteroni KF-1]
	57066	112 (63)	6.96	1.85	2.61	**25.59**	chaperonin GroEL [C. testosteroni CNB-2]
	43291	89 (59)	4.84	1.28	4.34	**26.01**	translation elongation factor Tu [C. testosteroni CNB-2]
	32521	40 (23)	4.75	1.26	0.19	**23.75**	extracellular solute-binding protein family 3 [C. testosteroni KF-1]
	14811	15 (10)	4.25	1.13	—	**25.17**	ribosomal protein L15 [C. testosteroni CNB-2]
	1154	16 (1)	4.25	1.13	—	**20**	RecName: Full = Quinoline 2-oxidoreductase gamma chain

^a^emPAI, (Exponentially Modified Protein Abundance Index). ^b^Charge, percent of charged amino acid.

## Discussion

### The reduction of selenite in *C. testosteroni* S44

The reduction of selenite is an effective detoxification process^6,7,40,41^, but the molecular mechanisms are still barely understood, especially in aerobic bacteria. According to previous studies, several genes such as *trxB*, *selD* and *selA* indicated the existence of Se assimilatory pathways^42–44^. Recently, a chromate reductase CsrF in *Alishewanella* sp. was shown to be essential for Se (IV) reduction to generate BioSeNPs *in vivo* and *in vitro* under aerobic condition^45^. In *C. testosteroni* S44, *trxB* encoding an enzyme catalyzing the transformation of selenite to selenopersulfide, *selD* encoding a selenophosphate synthetase and *selA* encoding a selenocysteine synthase were found on the genome. Therefore, the capability of both assimilatory and dissimilatory reduction of selenite may be present in *C. testosteroni* S44. It may present a detoxification process when the concentration of selenite reaches a certain threshold level. In strain S44, most of the BioSeNPs formation occurred in proximity of the cell border (Fig. 1D and E), which may be due to the demand of selenite reductase accepting electron from electron donors within the inner membrane.

### The coating organic agents especially proteins stabilize the BioSeNPs

The intracellular organic agents coating the surface of BioSeNPs were shown to play an essential role on the stability of SeNPs. TEM confirmed the presence of a thick layer of organic matter on BioSeNPs (Fig. 3A). When the coating agents were removed from BioSeNPs, the zeta potential of BioSeNPs decreased from −31.4 to −28.4 mV (Figs 1F and S2). And also, the SeNPs without surface organic matter were easier to precipitate on the bottom of the container than BioSeNPs, showing a loss of colloidal character and stabilization of BioSeNPs. Moreover, the smaller SeNPs aggregated into bigger ones after removing coating agents (Fig. 3C and D). Therefore, these coating agents played an essential role on the stability of BioSeNPs.

The FT-IR spectra showed the coating organics on BioSeNPs are proteins, carbohydrates and lipids (Fig. 4). The spectra were very similar between cellular fractions, BioSeNPs and CheBioSeNPs, and the shape of the FT-IR spectra of BioSeNPs was more closely related to the shape of FT-IR spectra of cellular fractions, indicating the features of BioSeNPs were correlated to the cellular fractions. The distinct shift of some spectral features such as at 1,400 and 3,440 cm^−1^ may be attributed to the interaction of organic matters with elemental selenium^26^. In contrast, the features of BioSeNPs and CheBioSeNPs differed from cellular fractions at 1,540 cm^−1^, which may due to the lower content of proteins compared to cellular fractions, the lower feature at 1,240 cm^−1^ reflected the presence of trace amounts of nucleic acid and phospholipids on BioSeNPs.

Quantitative detection showed the coating agents were mainly proteins, and a small quantity of carbohydrates, as well as limited amounts of lipids. It was clear that proteins play the major role on the formation of BioSeNPs in cells. Therefore, proteins are the major factors to govern the formation process and the stabilization of BioSeNPs, and the tiny amounts of carbohydrates and lipids play a minor role on this process.

### Nonspecific functional but charged amino acid enriched proteins assemble the selenium nanoparticles

Considering the protein profiles shown by SDS-PAGE (Fig. 5) are diverse and very similar between cellular fractions, BioSeNPs produced *in vivo* and CheBioSeNPs produced *in vitro*, proteins binding to BioSeNPs could be a physiochemical process in *C. testosteroni* S44, which would be in contrast to previous studies showing that the specific protein SefA helped assemble BioSeNPs in an anaerobic bacterium^33^. This point was confirmed by protein binding tests showing that single proteins strongly and randomly bound to BioSeNPs (Fig. 6). We quantified the amounts of coating proteins on the surface of BioSeNPs. In addition to the reported proteins in independent qualitative proteomic investigations, such as porin, chaperone protein, elongation factor, alcohol dehydrogenase, ribosomal protein and heat shock protein^28,31^, we confirmed that more proteins probably were involved in the formation of SeNPs in cells, including citrate synthase I, universal stress protein A (UspA), electron transfer flavoprotein and succinyl-CoA synthetase (more than 1% abundance). However, some abundant proteins (more than 1%) in cellular fractions, such as peroxiredoxin, extracellular solute-binding protein family 3 and Quinoline 2-oxidoreductase gamma chain, did not occur in the list of dominant coating-proteins (Table 1), i.e., coating proteins on BioSeNPs are not always correlated to the contents of proteins of cells. One reason, probably the most important, is the quantity of charge of amino acids (Asp, Glu, Arg and Lys) of proteins. The percentage of charged amino acids (Asp, Glu, Arg and Lys) for the proteins are shown in Table 1. In most cases, proteins containing more charged amino acids were better adsorbed to BioSeNPs despite of a lower content in cells. Therefore, when a few proteins have a lower abundance but containing a higher quantity of charged amino acids, then these proteins had a higher binding ability and a higher content on the surface of BioSeNPs, e.g. chaperone protein DnaK, elongation factor G, ribosomal protein S1 and heat shock protein 90. This is similar to results showing that amino acids with charged R groups were adsorbed more readily on minerals/clays/sediments than other amino acids^46^. However, the third predominant coating protein on BioSeNPs, zinc-dependent alcohol dehydrogenase (AdH) (Table 1), had a lower number of charged amino acids and cellular content. It is interesting that AdH was the dominant coating-protein on BioSeNPs as has occurred in many bacteria^30–32^. Accordingly, we have disrupted the gene encoding Adh, but could show that Adh was not essential for selenite reduction (data not shown). This does not rule out the likely possibility that Adh is involved in Se(0) reduction to Se(-II). Another possible reason is that the AdH bound on BioSeNPs with cysteine residues^47^. Other abundant proteins in cellular fractions such as peroxiredoxin did not appear in abundance as coating proteins due to their lower number of charged amino acids. Notably, this mechanism also elucidated the phenotype that not only one specific protein is involved in the formation of intracellular BioSeNPs in a microbial community^28^.

Another reason that coating proteins on BioSeNPs did not match the contents of cellular proteins is the place of BioSeNPs formation. In our case, the majority of BioSeNPs produced by *C. testosteroni* S44 were generated in the proximity of the inner membrane, either in periplasm or in the inside border of cell membrane (Fig. 1). Consequently, many cytoplasmic proteins became the dominantly coating proteins of BioSeNPs. In particularly, the cell membrane protein electron transfer flavoprotein beta-subunit (1.12% in abundance) coated on BioSeNPs, and this protein could be responsible for electron transformation to selenite reduction. The porin had a higher abundance both in this case and in previous report^28^ may due to BioSeNPs forming in periplasm to be transported to extracellular space via out membrane, or mature BioSeNPs to be released through cell lysis^24,28^. On the other hand, a few protein bands between CheBioSeNPs and BioSeNPs (Fig. 5, lane 1 and lane 3) were different, implying certain proteins bound to CheBioSeNPs but had no chance to bind BioSeNPs in cells because of spatial isolation.

Totally, the most important factors controlling the binding ability of the proteins on BioSeNPs in cells were percentage of charged amino acids, followed by spatial distribution of proteins. This research implies that certain proteins or single protein, even charged amino acids (Asp, Glu, Arg and Lys) could be used for artificial green synthesis of SeNPs and to control their formation and stability under simplified and temperate conditions, also it can help the surface modification of SeNPs for medicine application.

### Schematic model for the molecular mechanism of BioSeNPs formation in bacteria

**Figure 7:**
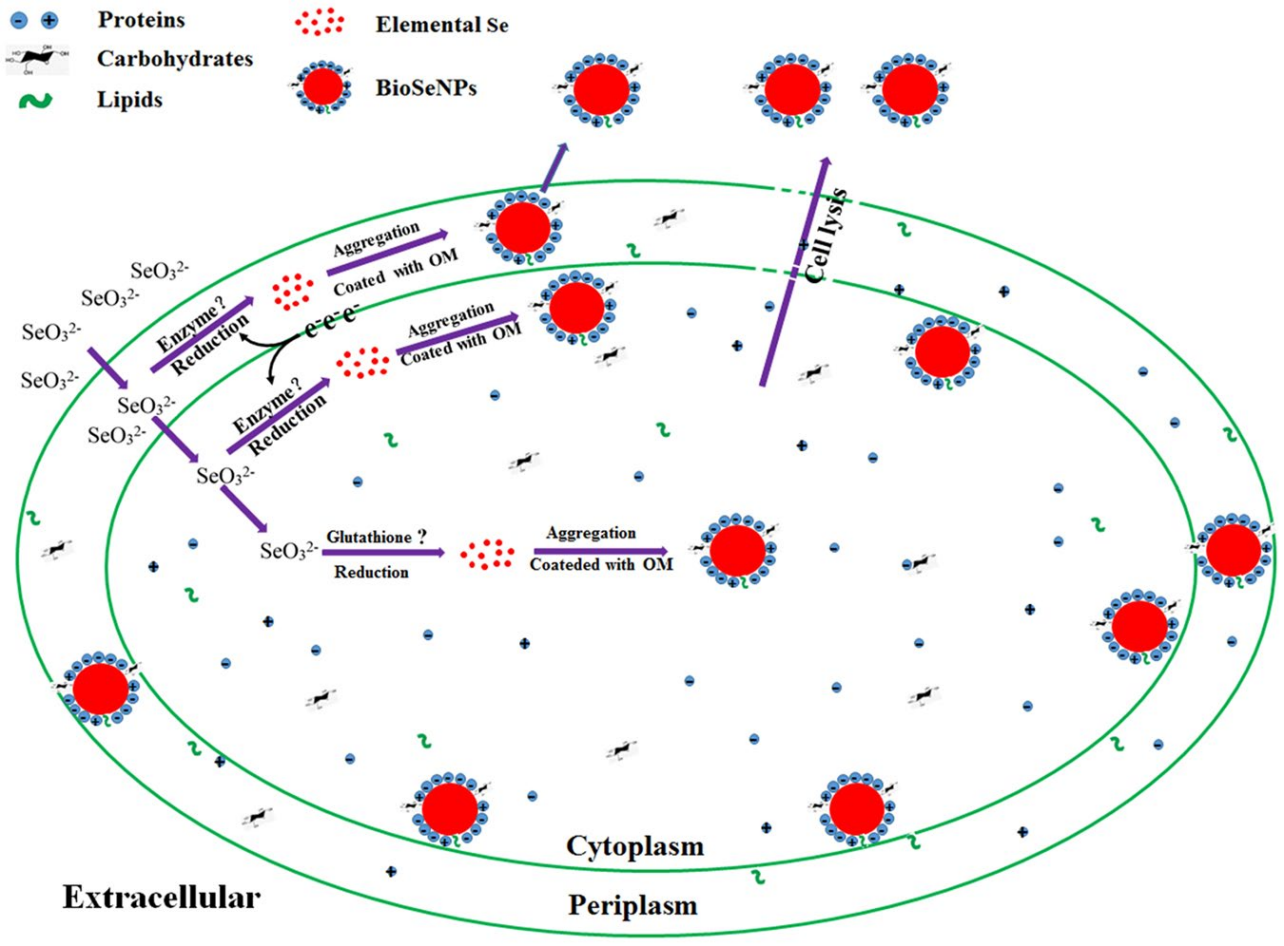
Schematic model for the molecular mechanism of BioSeNPs formation in bacteria. The majority of biosynthesized elemental selenium nanoparticles (BioSeNPs) are formed closing to the proximity of the cell border due to the demand of selenite reductase accepting electron from electron donors within the inner membrane. A little selenite is reduced by glutathione and thus BioSeNPs may appear in center of cells. During the total process of BioSeNP formation and stabilization in bacterial cells, proteins enriched in charged amino acids are the major factors, carbohydrates and lipids are the minor factors.

The formation mechanism of BioSeNPs in *C. testosteroni* S44 was summarized in Fig. 7. The selenite enters the periplasm and may be reduced by enzymes, such as nitrite reductase^48^ and fumarate reductase^49^. Other selenite is transported into cytoplasm and be reduced by reductases such as glutathione reductase^50^. The BioSeNPs occurred closing to the inner membrane may due to the demand of selenite reductase accepting electron from electron donors within inner membrane. A little selenite is reduced by glutathione and thus BioSeNPs may appear in center of cells^51^. Then elemental selenium aggregates gradually and is coated by organic matters (OM). The mature BioSeNPs may be released through cell lysis. During the total process of BioSeNP formation and stabilization in cells, proteins enriched in charged amino acids are the major factors, carbohydrates and lipids are the minor factors.

## Conclusions

BioSeNPs were produced by selenite reduction both in the proximity of the cell border (majority) and cytoplasm (minor) in the aerobic bacterium *C. testosteroni* S44. Nonspecific functional but charged amino acid enriched proteins are the major factors to govern the formation process and the stabilization of BioSeNPs. In contrast, the tiny amounts of carbohydrates and lipids played a minor role in this process. The findings are helpful to guide the artificial green production of SeNPs by certain proteins under temperate and simplified conditions and help the surface modification of SeNPs for medicine application in the future.

## Methods

### Production and purification of bio-synthesized elemental selenium nanoparticles (BioSeNPs)

A 1% *Comamonas testosteroni* S44 inoculum was incubated in 250 mL Erlenmeyer flasks containing 100 mL Luria-Bertani (LB, Difco) broth and cultured for 12 h (up to the middle of exponential growth), then 10 mM sodium selenite was added and incubation continued at 28 °C with shaking at 150 rpm for 3 days. The production of elemental selenium was confirmed by the appearance of red color. The culture was collected by centrifugation (Eppendorf 12492) for 10 min at 8,000 rpm. Cells were lysed by sonication after washing twice by double distilled water (ddH_2_O, 18.25 MΩ·cm). Following centrifugation (Eppendorf 5415D) at 12,000 rpm for 5 min and removing the supernatant, the pellets were then resuspended in ddH_2_O and centrifuged twice with 80% (w/v) sucrose for 30 min to remove biomass (Fig. S1). The pure BioSeNPs on the bottom were collected after washing twice by ddH_2_O and temporarily preserving in −20 °C.

### Production of selenium nanoparticles (CheBioSeNPs) by L-reduced glutathione (GSH)

The production of CheBioSeNPs followed the protocol developed by Jain *et al*.^26^ with minor modifications. A 1% inoculum of *C. testosteroni* S44 was inoculated in 250 mL Erlenmeyer flasks containing 100 mL LB broth and cultured for 3.5 days. Cells were collected by centrifugation (Eppendorf 5810 R, 8,000 rpm, 4 °C) and washed twice with ddH_2_O. Then, bacterial cultures were concentrated in 20 mL ddH_2_O and lysed by French pressure, the supernatant of lysed cellular fractions (SLCF) was collected after centrifugation (Eppendorf 5810 R, 8,000 rpm, 4 °C, 30 min). CheBioSeNPs were generated by adding 200 µL 1 M sodium selenite and 0.25 g GSH into the SLCF at room temperature. Pure CheBioSeNPs were collected by centrifugation (Eppendorf 5415D, 12,000 rpm, 15 min) after washing twice by ddH_2_O.

### Transmission Electron Microscopy (TEM) and Electron Dispersion X-ray Detector (EDX) analyses

*C. testosteroni* S44 was cultured in LB broth for 3 days with the addition of 10 mM sodium selenite. Collected cells were centrifuged (Eppendorf 5810 R) at 4,000 rpm and washed twice by 0.85% sodium chloride solution. The cells were immobilized with 2.5% glutaraldehyde overnight and then rinsed three times in 0.15 M sodium cacodylated buffer (pH 7.2) for 2 h. The specimens were dehydrated in graded series of ethanol (15, 30, 50, 75, 95 and 100%) and embedded in Epon for preparation of sections. The sections were collected on copper grids with Formvar supporting membranes. Images were obtained with Hitachi H-7650 (Japan) after staining with uranyl acetate. The ultrathin sections for TEM and EDX were obtained by a cryosection system (UC6, Leica, Germany).

### The Dynamic Light Scattering (DLS), zeta potential and Fourier Transform Infrared (FT-IR) spectroscopy analysis of SeNPs

BioSeNPs and CheBioSeNPs were suspended in ddH_2_O. Absorbance was measured using a visible light spectrophotometer at wavelengths between 350 to 900 nm. Dynamic Light Scattering (DLS) and zeta potential analyses were performed using Mastersizer 2000 (UK). The lyophilized BioSeNPs, CheBioSeNPs and SLCF from *C. testosteroni* S44 were prepared for fourier transform infrared (FT-IR) spectroscopy analysis. The sample was mixed with spectroscopic grade potassium bromide (KBr, dried for 24 h at 60 °C) in a ratio of 1:100 and the spectrum recorded in the range of 400–4000 wavenumber (cm^−1^) on the FT-IR spectrometer, Spectrum 100 (Perkin Elmer, USA) in the diffuse reflectance mode at a resolution of 4 cm^−1^ in KBr pellets.

### Quantification of Se and chemical composition of coated agents on BioSeNPs

The Se concentration was determined by the method of Keka *et al*.^52^. The carbohydrates were determined as described Jain *et al*.^26^ with minor modifications. Purified BioSeNPs were suspended in ddH_2_O and sonicated for 30 min, then held in a boiling water bath for 10 min to release as many carbohydrates as possible. The supernatant was used for quantification of carbohydrates^53^ after centrifugation (Eppendorf 5415D, 12,000 rpm, 5 min). Protein quantification was performed using the Lowry assay^54^. BioSeNPs were suspended in 10% SDS solution and held in a boiling water bath for 20 min to strip the binding proteins. The supernatant was used for protein quantification after centrifugation (Eppendorf 5415D, 12,000 rpm, 5 min). Lipid extraction and determination of BioSeNPs were used the method described by Drenovsky *et al*.^55^.

### Proteins analyzed by SDS-PAGE and proteomics

Binding proteins striped from BioSeNPs and CheBioSeNPs were used the method described by Dobias *et al*.^30^ with minor modifications. Pure SeNPs was mixed in 10% sodium dodecyl sulfate (SDS) solution and held in a boiling water bath for 20 min to separate the binding proteins. The stripping proteins were characterized by SDS-polyacrylamide gel electrophoresis (SDS-PAGE) techniques^56^. The proteins from gel were purified and digested based on the protocol described by Katayama *et al*.^57^, digested peptide mixtures were analyzed by liquid chromatograph-mass separation-mass spectra (LC-MS-MS, by Sangon, Shanghai, China). Protein identification was performed by searching the National Center for Biotechnology Information non-redundant database (NCBInr) using the Mascot Program (http://www.matrixscience.com).

## Electronic supplementary material

Supplementary Information

Dataset 1

Dataset 2
