# Supplementary material for: Proteins enriched in charged amino acids control the formation and stabilization of selenium nanoparticles in Comamonas testosteroni S44
Source: Sci Rep. 2018 Mar 19;8:4766. doi: 10.1038/s41598-018-23295-5 (PMC5859168; doi:10.1038/s41598-018-23295-5)
Supplement: Supplementary file 1 — Supplementary Information [file 41598_2018_23295_MOESM1_ESM.pdf]

Proteins enriched in charged amino acids control the formation and stabilization of selenium nanoparticles in *Comamonas testosteroni* S44

Ding Xu[a], Lichen Yang[a], Yu Wang[a], Gejiao Wang[a], Christopher Rensing[b] and Shixue Zheng[*a]

[a]State Key Laboratory of Agricultural Microbiology, College of Life Science and Technology, Huazhong Agricultural University, Wuhan, 430070, P. R. China

[b]Institute of Environmental Microbiology, College of Resources and Environment, Fujian Agriculture & Forestry University, Fuzhou, Fujian 350002, P. R. China

*** Corresponding author:**

Shixue Zheng

Tel: +86-27-87294148; Fax: +86-27-87280670

E-mail: zhengsx@mail.hzau.edu.cn

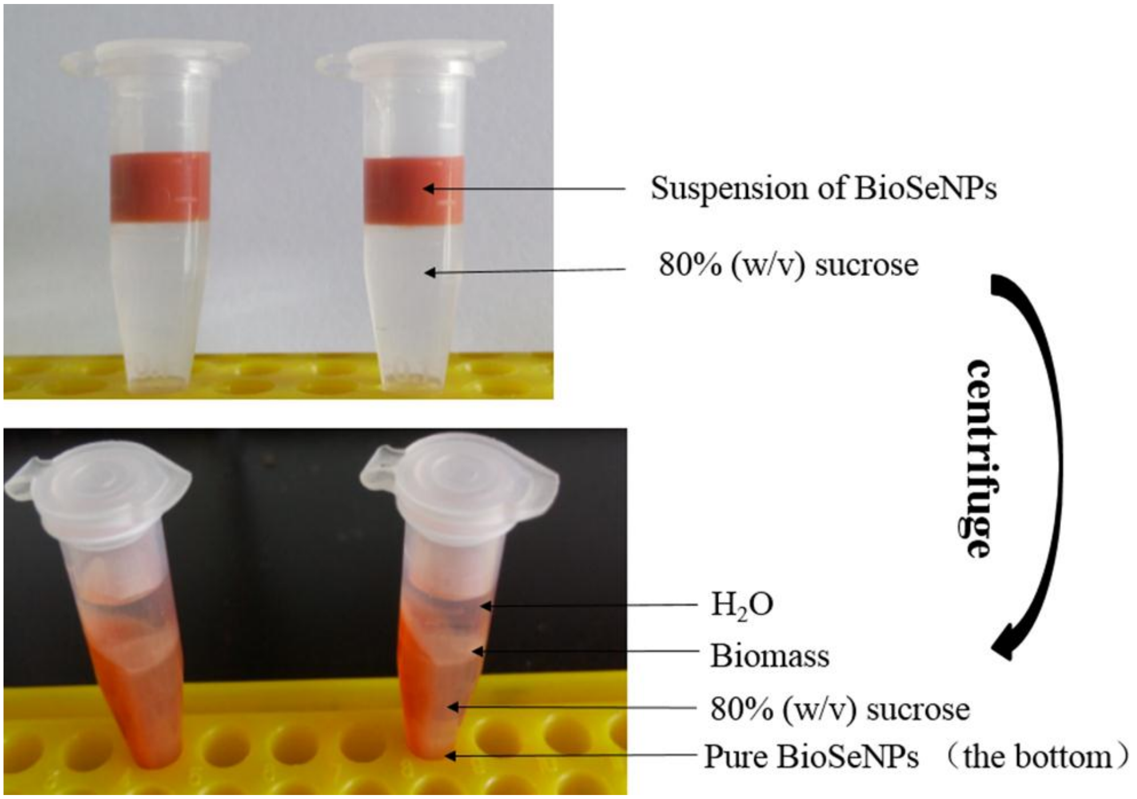

Fig. S1. Method of BioSeNPs purification

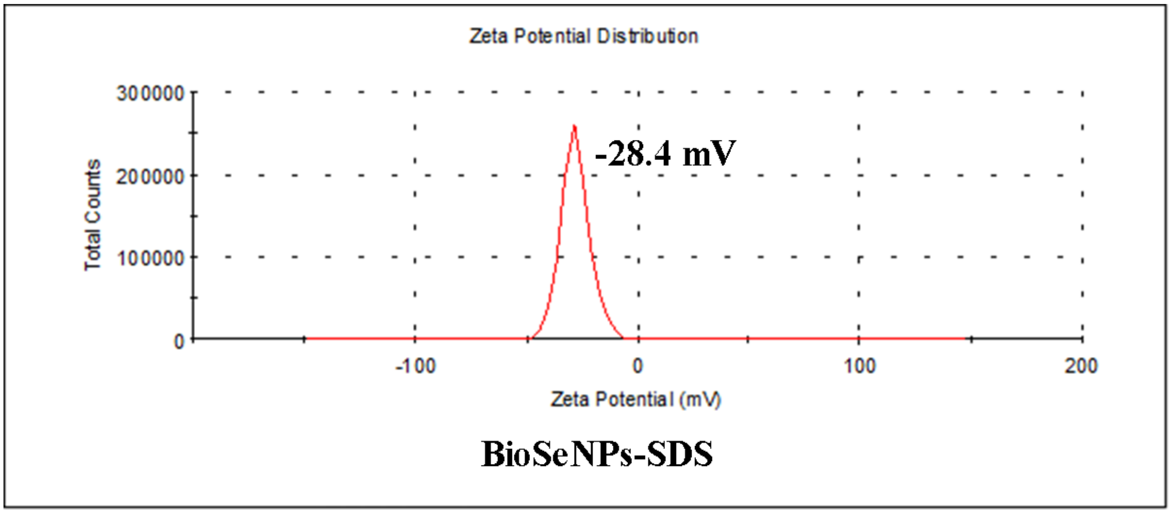

Fig. S2. The zeta potential of BioSeNPs after the coating agents were almost removed by 10 % SDS solution.

Method

LC-MS/MS Analysis

Samples were re-suspended with Nano-RPLC buffer A (Sangon, Shanghai, China). The online Nano-RPLC was employed on the Eksigent nanoLC-Ultra™ 2D System (AB SCIEX). The samples were loaded on C18 nanoLC trap column(100 µm ×3cm, C_{18}, 3 µm, 150Å) and washed by Nano-RPLC Buffer A (0.1%FA, 2%ACN) at 2µL/min for 10 mins. An elution gradient of 5-35% acetonitrile (0.1%formic acid) in 90 mins gradient was used on an analytical ChromXP C18 column (75µm x 15 cm, C18, 3µm 120 Å) with spray tip. Data acquisition was performed with a Triple TOF 5600 System (AB SCIEX, USA) fitted with a Nanospray III source (AB SCIEX, USA) and a pulled quartz tip as the emitter (New

Objectives, USA). Data were acquired using an ion spray voltage of 2.5 kV, curtain gas of 30 PSI, nebulizer gas of 5 PSI, and an interface heater temperature of 150 ℃. For information dependant acquistion (IDA), survey scans were acquired in 250 ms and as many as 35 product ion scans were collected if they exceeded a threshold of 150 counts per second (counts/s) with a 2^+ to 5^+ charge-state. The total cycle time was fixed to 2.5s. A rolling collision energy setting was applied to all precursor ions for collision-induced dissociation (CID). Dynamic exclusion was set for ½ of peak width (18 s). And the precursor was then refreshed off the exclusion list. Based on combined MS and MS/MS spectra, proteins were successfully identified based on 95% or higher confidence interval of their scores in the MASCOT V2.3 search engine (Matrix Science Ltd., London, U.K.), using the following search parameters: cow-lacbobacillus casel mix database, trypsin as the digestion enzyme, two missed cleavage site, fixed modifications of Carbamidomethyl (C), partial modifications of Acetyl (Protein N-term), Deamidated (NQ), Dioxidation (W), Oxidation (M) Phospho (ST) and Phospho (Y). ±15ppm for precursor ion tolerance and ±0.15Da for fragment ion tolerance.
